# Choice Extract

**Published:** 1850-04

**Authors:** 


### THE MEDICAL EXAMINER.

PHILADELPHIA, APRIL, 1850.

The following choice extract from a distant country paper* was sent to us by a friend for our perusal. It is too good to be entirely lost, and we therefore present it to our readers, albeit slightly shortened in its fair proportions. It would seem that the sovereign people are beginning to be awake to their "doctoring" necessities, and evince a laudable desire to diminish the want above expressed by Dr. Mitchell, by filling up the vacuum at the shortest notice and with such material as they have at hand.

*Milledgeville*, Feb. 13, 1850.

* * * * But let us go back a few days, to Saturday last, which, if I mistake not, was the 9th, and into the other branch where dignity is only disrespected—when dignity would be a bore. The bills of the House of the first and second reading had been discharged, and the Secretary had taken another order. Bills which had been committed to the Committee of the whole, and in the course of the order, one was taken up "to license one Stephens, of Gilmore County, to practice Medicine without license." Dr. J. R. Smith, when the bill was read, moved to postpone it indefinitely—he was opposed in principle to the enactment of any law of the kind; under the present general law men were required to familiarize themselves with diseases and reme-

* The Daily Constitutionalist, Augusta, Ga.

dies, with the general rules of the science, and undergo an examination by a board of experienced and erudite Physicians appointed for that particular purpose, who should pass upon their qualifications, before they were allowed to manœuvre with the delicate machinery of man's constitution. Health was a jewel beyond all estimation, and the laws had wisely guarded it as far as law could, from the injuries of empyricism; and that this bill proposed an injustice to that class of men who had devoted time and treasure to prepare for the responsible duties of a physician. It would bring in competition which would degrade the profession and lower the standard of excellence by which that class were wont to be judged; and compromise the position which the people had a right to expect the Legislature would give to those who were to minister to their infirmities. That no man should be allowed to put himself upon a community as skilled in medicine, unless he had been found so, as by the very act he might, in critical cases, be trusted to the ruin and death of worthy and excellent citizens. He considered the measure inexpedient, as it inevitably would establish a precedent likely to defeat the wise ends which the present law proposes to accomplish, and open the way to endless petitions of a like character. We cannot pursue the argument further, as it is alien to the purpose of this letter, but we consider his views sound and very appropriate.

Mr. Edmondson agreed with the Senator from the 21st, but hoped his motion would not prevail, as the people in his section were anxious for the passage of the bill, and he felt inclined to defer to their wishes. It was entirely local in its character, and he believed the applicant to be a worthy citizen of the county; he knew nothing of his qualifications, but hoped the pleasure of his constituents would be consulted in this case. The motion to postpone was lost.

Mr. James R. Smith moved to amend by adding, as an additional section, that all laws requiring applicants to undergo examination in order to practice, be abolished.

Mr. James E. Brown moved to amend the bill by adding the following, to wit: Be it further enacted, that William Henry and Francis Singleton, of Tatnall county, be authorized to practise physic under the restrictions and rules contained in this act.

Mr. Anderson moved to amend by the following: That the persons herein named pay the State and county tax usually imposed on Physicians in this State. Another Senator moved to amend by adding the following proviso: That the passage of this act shall not be considered a precedent to authorize the Senate of Georgia to resolve itself into a Medical Faculty, in future, to manufacture Physicians without regard to qualifications, or the necessary requisitions under the laws of the State.

Mr. Ferrel also moved the additional proviso: That the said Stephens and others shall not administer the steam bath more than three times to any patient in 24 hours, nor give a dose of Wake-Robin until the patient has been dead at least 12 hours, nor Mountain Root, only in extreme cases.

Mr. McBee hoped that *Sinators* would not stick on so many amendments likely to injure the bill.

Mr. William Jones offered an amendment which he wanted to lay upon the table to the effect—That the Honourable degree of M. D. be conferred upon the Senator from the 46th District, and he be allowed to administer *Roots* to his patients, if so desired, and in each and every case be entitled to exact the fee usually allowed in such cases.

Mr. McBee, then rose and made the following speech, to wit:

[The speech of the gentleman from the 46th District, which should follow in this place, is so *killing* to the whole profession, and so destructive to the mother tongue, that we feel bound to spare our readers its recital.—Ed. Ex.]

On the passage of the bill the vote stood 22 yeas to 14 nays.

With such a brilliant exhibition of legislative wisdom to enlighten us we cannot close our eyes to the lamentable fact that there really is a scarcity of doctors somewhere. However lightly this sort of famine may weigh upon us here at the fountain head of a large proportion of American medical existence, still less will this sapient history allow us to presume that, in one sovereign state at least, the call for aid in the doctor-making business will not be promptly answered.

Verily the invention of these honorables is worthy of a much higher latitude, and bids fair to assist in releasing our schools from a deal of anxious struggling for supremacy in catalogue expansion. The Gordian knot which has so puzzled and excited our National Association is cut at last, without either scalpel or prescription for its *trenchant.* Henceforth the glories of the lecture room, the dissecting table and the hospital, are departed—the professor's occupation is gone, and long courses, short courses, and biennial courses are alike doomed to pass away unheeded and forgotten. Introductories, valedictories, examinations and commencements, the whole cumbrous machinery with which laborious instruction was wont to do the duty, may now be left for the free and easy route of intuition and a vote of the majority. All must change their once golden brightness for a melancholy glimmering among the things that were.

We have changed all that. The ordeal of the green-box must give way to the cringes of the lobby member,—committees of the whole take the chairs of the faculty as they do the benches of the court, and graduations and divorces move on together through the same unhallowed license.

To treat the matter seriously for a moment—which is more respect than we feel inclined to pay to such culpable absurdity—we have long had reason to suspect from personal observation, without the necessity of any additional conviction arising from the action chronicled in the Georgia letter, that there is a sad deficiency of legitimate practitioners in many, very many portions of our Union. We are satisfied that the whole number of *bonâ fide* graduates is not only unequally distributed, but that it is so entirely inadequate to the wants of the community, that pretenders and incompetent physicians are necessarily encouraged and fostered to an extent that would justify, for the removal of the evil, the annual production of twice as many educated members of the profession as are now presented by our schools. Under this assurance we shall hail with pleasure the establishment of every new school, and rejoice in the increased prosperity of all the institutions now effectively at work, no matter how appalling the crowd of new candidates for medical honors and emoluments may seem to be. The deserving man may rely upon eventual success, if he be willing to seek it in the proper place. The undeserving need not be feared or pitied by any but himself, for he too shall have his reward.

